# optima: an open-source R package for the Tapestri platform for integrative single cell multiomics data analysis

**DOI:** 10.1093/bioinformatics/btad611

**Published:** 2023-10-05

**Authors:** Dong Pei, Rachel Griffard, Nanda Kumar Yellapu, Emily Nissen, Devin C Koestler

**Affiliations:** Department of Biostatistics & Data Science, University of Kansas Medical Center, Kansas City, KS 66160, United States; The University of Kansas Cancer Center, Kansas City, KS 66160, United States; Department of Biostatistics & Data Science, University of Kansas Medical Center, Kansas City, KS 66160, United States; Department of Biostatistics & Data Science, University of Kansas Medical Center, Kansas City, KS 66160, United States; The University of Kansas Cancer Center, Kansas City, KS 66160, United States; Department of Biostatistics & Data Science, University of Kansas Medical Center, Kansas City, KS 66160, United States; Department of Biostatistics & Data Science, University of Kansas Medical Center, Kansas City, KS 66160, United States; The University of Kansas Cancer Center, Kansas City, KS 66160, United States

## Abstract

**Summary:**

The Tapestri platform offers DNA and protein analysis at the single-cell level. Integrating both types of data is beneficial for studying multiple cell populations in heterogeneous microenvironments, such as tumor tissues. Here, we present optima, an R package for the processing and analysis of data generated from the Tapestri platform. This package provides streamlined functionality for raw data filtering, integration, normalization, transformation, and visualization. Insights gained from the optima package help users to identify unique cell populations and uncover surface protein expression patterns. The results generated by optima help researchers elucidate dynamic changes at the single-cell level in heterogeneous microenvironments.

**Availability and implementation:**

This package is available in Github: https://github.com/rachelgriffard/optima.

## 1 Introduction

Recent advances in molecular barcoding make it possible to perform next-generation transcriptomic sequencing at the single-cell level, such as 10× Genomics Chromium (Zheng *et al.* 2017) and DropSeq (Macosko *et al.* 2015). Further, the emergence of CITE-seq allows simultaneous profiling of surface protein on top of transcriptome profiling for the individual cells (Stoeckius *et al.* 2017). Meanwhile, for characterizing DNA and protein profiles, a platform called Tapestri was launched in 2017 (Mission Bio, South San Francisco, CA, USA). This platform analyzes a predefined panel of DNA variations and cell surface proteins, simultaneously. By utilizing the data collected from the Tapestri platform, researchers can identify unique cell populations within a heterogeneous tissue. This platform has been used widely in blood cancer studies (McMahon *et al.* 2019, Demaree *et al.* 2021, Peretz *et al.* 2021) for charactering cell clones within patients. It has also been used to study other types of human malignancies, such as colon cancer (Gao *et al.* 2019), as well as non-cancer settings that involve resolution of heterogeneous cell populations (Hacken *et al.* 2020).

**Figure 1.**
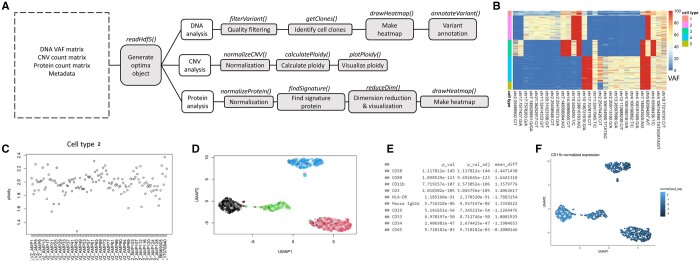
(**A**) Potential pipeline for analysis of single cell multiomic data from the Tapestri platform. Boxes represent the different steps of the analysis pipeline, accompanied by related function names above each box. (**B**) Example heatmap for DNA data (VAF). The rows in the heatmap was sorted by the cell clones identified from function getClones(). (**C**) Scatter plot of ploidy for each CNV amplicon in cell clones. (**D**) Dimension reduction plot of the cells using reduceDim() function using protein expression data. Each cell was colored by cell clone label identified in previous step. (**E**) Result table for top signature proteins in cell type “1” generated by findSignature() function. (**F**) Visualization of one protein feature, CD11b, with colored overlay in a dimension reduction plot.

To process CITEseq data that contains single-cell RNA and protein data, several open-source packages can be used, including Seurat (Hao *et al.* 2021), CITEfuse (Kim *et al.* 2020), iCluster (Shen *et al.* 2009), iCluster+ (Mo and Shen 2023), etc. To preprocess raw sequencing data in the Tapestri platform, the Tapestri Pipeline software (https://portal.missionbio.com/, Mission Bio, South San Francisco, CA, USA) had been developed for generating variant calls and protein expression counts. For downstream analysis, a Python library mosaic (https://missionbio.github.io/mosaic/) has been developed by Mission Bio. In addition, there is a GUI software called Tapestri Insight that supports Tapestri DNA data analysis. However, Tapestri insight is limited in that it does not support protein data analysis. While the R script used for analyzing Tapestri platform data in studying myeloid malignancies (Miles *et al.* 2020) is freely available on GitHub (https://github.com/bowmanr/scDNA_myeloid), to our knowledge, there is yet no R package for the processing and analysis of molecular data generated from the Tapestri platform. Here, we present optima, an Open-source R Package for the Tapestri platform for Integrative single-cell Multiomics data Analysis. Optima is designed to simplify the workflow for the preprocessing, analysis, and visualization of multiomic data generated by the Tapestri platform (Fig. 1A).

## 2 Implementation

### 2.1 Input data

The core object in the optima package is the optima object. This object stores all data matrices for a single biological sample, including DNA (amplicon sequencing data for DNA variants), CNV (copy number variation), and protein. This object also stores all the metadata, including cell barcodes, panels of amplicon names, as well as metadata to keep track of normalization/filter status, etc. When called directly, this object displays summary statistics for the object. After initial preprocessing by the Tapestri pipeline software, an .h5 file will be generated and should be used as input file for the optima package. This file can be read into R environment as an optima object by using readHdf5() function.

### 2.2 DNA

Once an optima object is created, we begin by analyzing the DNA sequencing data. DNA analysis focuses on variant allele frequency for single nucleotide variants. The first step is DNA variant data filtering with the filterVariant() function. Several factors, including sequencing depth, genotype quality, etc., are imported from the .h5 file and used in this filtering step. A cell/variant will be removed if too many loci fail QC. If desired, users may supply their own parameters to achieve different QC thresholds instead of using the default values. After filtering, the DNA data will be used for cell clone identification. To identify clones, a user may choose to use the non-supervised clustering method dbscan (Hahsler *et al.* 2019) in the getClones() function. The clustering result will be stored in the cell labels vector contained within the optima object. If the user prefers to use domain knowledge to manually assign cell labels, they may do so by generating and assigning their own label to the cell label vector within the optima object. To visualize variant allele frequency in a heatmap, users can use the drawHeatmap() function. Heatmaps are generated by first sorting rows based on cell labels. To visualize variant allele frequency for one single variant in different cells, users may use the plotVariantFeature() function. Color overlay based on VAF will be generated on a dimension reduction plot. If the user is interested in obtaining annotation for the variants, the annotateVariant() function can be used. By fetching data from MissionBio’s API, this function takes all the variant ID’s as an input and returns an R data.frame containing detailed information for the variants.

### 2.3 CNV

After analysis of DNA sequencing data, the next step is the analysis of CNV. The major goal in the analysis of CNV data is to calculate the ploidy for each CNV amplicon. The input is based on the number/count of aligned read for each CNV locus. To correct for column-wise and row-wise variations in CNV counts, users can use the normalizeCNV() function. After normalization, users can calculate ploidy using the calculatePloidy() function. This is done by first defining a reference cell type as a diploid cell, then using the normalized count for other cell types, divided by the mean count of each CNV amplicon. The numeric values represent ploidy for each amplicon, in each cell.

### 2.4 Protein

In addition to DNA sequencing data, The Tapestri platform also quantifies cell surface proteins. The raw data for protein are generated as the counts of antibody binds to a specific surface protein. To process such data, optima uses the centered log-ratio transformation (Van Den Boogaart and Tolosana-Delgado 2008) method within the normalizeProtein() function. After data transformation, users may choose to visualize cells in a 2D space using dimension reduction methods with the reduceDim() function. In addition, users may visualize all normalized protein counts in a heatmap with the drawHeatmap() function. To visualize normalized protein expression for one single protein in different cells, users could use the plotProteinFeature() function. Color overlay based on normalized protein count will be generated on a dimension reduction plot. Lastly, by using the cell labels and the findSignature() function, users can identify proteins that are expressed differently in one cell type compared with all other cell types. Such comparison is done using a t-test. The result is an R data.frame that contains all proteins sorted by the smallest *P*-value after adjustment for multiple comparisons using the Benjamini–Hochberg FDR method.

## 3 Usage example

To illustrate the optima package, we applied it to an example dataset containing a four cell mixture. The dataset is in .h5 file format. As stated above, one optima object stores all data matrices for one single biological sample. In this example dataset, four cells were mixed as one biological sample. After being imported into R with readHdf5() function, this dataset is stored as an optima object. When called, this object displays summary statistics. More specifically, it contains 1313 cells, 27 719 variants, 127 CNVs, and 10 proteins. Users may supply their own .h5 file generated from their Tapestri pipeline output. Using the four cell mixture example, we performed filtering with the filterVariant() function. We started with 1313 cells and, after filtering, 1271 cells remain. Meanwhile, 29 variants were kept after filtering. After cell clone identification using the getClones() function, we identified six clusters/cell clones within the dataset. Each cell clone is labelled with numeric value 1, 2, 3, 4, 5, and 6 in the cell labels vector in the optima object. Such information is then used to generate a heatmap using the DNA sequencing data (Fig. 1B). For CNV analysis, we calculated the normalized counts with normalizeCNV() function and calculated ploidy with the calculatePloidy() function. With respect to the application of the calculatePloidy() function, cell type “1” was set as the diploid cell type. For visualization of the ploidy in each amplicon, we generated scatter plots (Fig. 1C). All cell type 1 amplicons have ploidy value at 2. This is because cell type 1 was specified as the diploid cell. For cell type 2, the ploidy ranges between 0 and 3. The protein analysis started with data normalization using normalizeProtein() function. The normalized protein counts were then used for projecting cells in a 2D space, and each data point was colored based on their cell type label. Upon visual examination of Fig. 1D, it is evident that there exist four prominent clusters, each exhibiting a predominance of points with identical coloration, signifying a consistent cell type assignment within each respective cluster. By comparing the protein expression level of one cell type against other cell types, the findSignature() function returns a table of differentially expressed proteins with p-value adjustments for multiple comparisons (Fig. 1E). To visualize expression level for “CD11b” protein, the plotProteinFeature() function was used. This function returns a scatter plot with all cells projected in 2D space. Each cell was colored based on CD11b protein expression level (Fig. 1F).

## 4 Conclusion

In conclusion, we developed optima, the first R package for the preprocessing and analysis of data generated from the Tapestri platform. The optima package has intuitive functionality for QC filtering, explorative analysis, and visualization. The DNA sequencing data are essential for identifying unique cell types within a heterogeneous environment. For example, different cell clones within cancer tissue. In addition, by leveraging the cell type data within a sample, optima allow users to investigate the difference of CNVs and protein expression levels among different cell types.

## Data Availability

The four cell mix dataset and metadata can be found from: https://portal.missionbio.com/datasets/4-cell-lines-AML-multiomics.
